# Low aspartate aminotransferase/alanine aminotransferase (DeRitis) ratio assists in predicting diabetes in Chinese population

**DOI:** 10.3389/fpubh.2022.1049804

**Published:** 2022-11-02

**Authors:** Wangcheng Xie, Weidi Yu, Shanshan Chen, Zhilong Ma, Tingsong Yang, Zhenshun Song

**Affiliations:** ^1^Department of General Surgery, Shanghai Tenth People's Hospital, Tongji University School of Medicine, Shanghai, China; ^2^College of Traditional Chinese Medicine, Southern Medical University, Guangzhou, China; ^3^Department of Pancreatic Surgery, Fudan University Shanghai Cancer Center, Shanghai, China

**Keywords:** alanine aminotransferase, aspartate aminotransferase, Chinese, diabetes, non-linearity

## Abstract

**Background:**

Few studies discussed the predictive ability of aspartate aminotransferase/alanine aminotransferase (AST/ALT, DeRitis) ratio for diabetes risk. The aim of this study was to characterize the role of AST/ALT ratio in the prediction of Chinese diabetes.

**Methods:**

This retrospective cohort study analyzed a Chinese population comprising 87,883 participants without diabetes at baseline between 2010 and 2016. Cox proportional hazards regression was used to identify independent risk factors. Restricted cubic spline (RCS) was performed to investigate the non-linear correlation between AST/ALT ratio and diabetes risk.

**Results:**

During a median follow-up period of 3.01 years, 1,877 participants developed diabetes. Comparing the baseline characteristics, diabetes group exhibited lower AST/ALT ratio. The Kaplan-Meier curve showed that participants with low AST/ALT ratio had higher cumulative incidence, and Cox regression also demonstrated that the lower AST/ALT ratio, the higher diabetes risk (HR: 0.56, 95% CI: 0.37–0.85, *P* = 0.006). The RCS model revealed a non-linear correlation between AST/ALT ratio and diabetes risk. In the condition of AST/ALT ratio ≤1.18, diabetes risk increased as it decreased (HR: 0.42, 95% CI: 0.19–0.91, *P* = 0.028). In contrast, AST/ALT ratio did not independently affect diabetes when beyond 1.18.

**Conclusion:**

AST/ALT ratio is a valuable predictor of diabetes. Diabetes risk increases rapidly in the condition of AST/ALT ratio ≤1.18.

## Introduction

Diabetes is a common disease that has become a major public health challenge worldwide as its prevalence has been increasing in recent years (1). It is estimated that 537 million adults currently have diabetes in the world, and the number is expected to rise to 783 million by 2045, among which more than 90% are type 2 diabetes (2). In 2021, China had approximately 145 million diabetic adults, more than any other country around the world, and the overall prevalence reached 10.6% (3). Diabetes and concomitant complications not only affect the health of the patients, but also cause a huge economic pressure on the patients and the healthcare system (4). Therefore, it is particularly important to screen for predictors of diabetes for early prevention and management of high-risk populations.

Alanine aminotransferase (ALT) aspartate aminotransferase (AST) and gamma-glutamyltransferase (GGT) have been identified as markers of liver function, and liver dysfunction has been implicated in diabetes (5). Numerous studies have pointed to the potential of ALT, AST and GGT values to predict the occurrence of diabetes, however, the results obtained in different populations are variable (6–8). The AST/ALT (DeRitis) ratio was first introduced in 1957 and was primarily used to assess the severity of viral hepatitis (9). In recent years, an increasing number of researches have elucidated its capacity to act as a predictor for other diseases such as non-alcoholic fatty liver disease (NAFLD), metabolic syndrome, peripheral arterial disease and chronic kidney disease (10–13). At the same time, it has been reported that high ALT/AST ratio are strongly associated with insulin resistance and insulin resistance-related diseases in the Korean population (14). Further studies have shown that a low AST/ALT ratio is an ideal predictor of the incidence of diabetes in Japanese (15, 16).

However, there is still a lack of studies investigating the efficiency of the AST/ALT (DeRitis) ratio in predicting the occurrence and progression of diabetes in Chinese population. Therefore, the aim of this study was to evaluate the exact association between AST/ALT ratio and diabetes by utilizing and analyzing a large retrospective cohort study in China.

## Methods

### Participants

**Figure 1:**
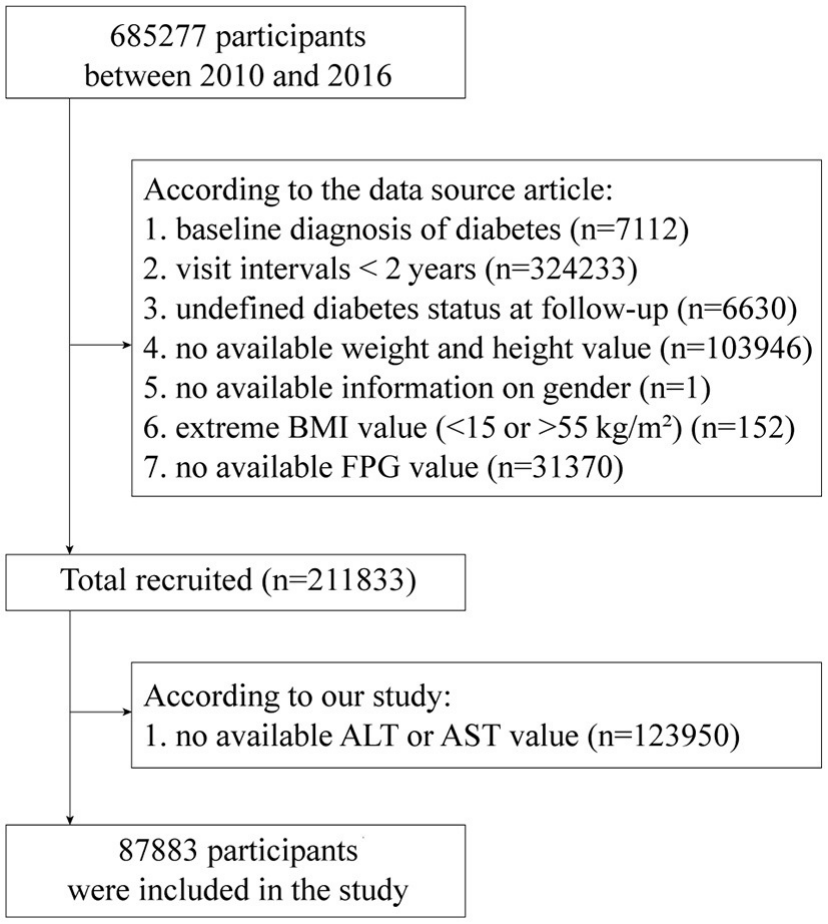
The flow chart of study participant selection. ALT, alanine aminotransferase; AST, aspartate transaminase; BMI, body mass index; FPG, fasting plasma glucose.

The original data for this study was obtained from a publicly available dataset within the Dryad Digital Repository (https://doi.org/10.5061/dryad.ft8750v) (17, 18). The initial study cohort included a total of 685,277 participants from 11 cities in China, who were at least 20 years old with at least 2 visits between 2010 and 2016. According to Chen's study design, participants who met the following criteria had been excluded: baseline diagnosis of diabetes (*n* = 7,112), visit intervals <2 years (*n* = 324,233), undefined diabetes status at follow-up (*n* = 6,630), no available weight and height value (*n* = 103,946), no available information on gender (*n* = 1), extreme body mass index (BMI) value (<15 kg/m^2^ or >55 kg/m^2^) (*n* = 152) or no available fasting plasma glucose (FPG) value (*n* = 31,370) (17). Thus, the original dataset we obtained contained 211,833 participants. We then excluded 123,950 participants without available ALT or AST value. Finally, a total of 87,883 participants were recruited into our study cohort (Figure 1).

The primary outcome indicator for this study was incident diabetes, defined as FPG ≥7.00 mmol/L and/or self-reported diabetes during the follow-up period (17). In addition, specific measurements for other indicators including demographics, lifestyle, chronic disease history, physical examination, and laboratory tests were reported in detail in previous studies (17).

This study obtained ethical exemption from the Ethics Committee of Shanghai Tenth People's Hospital because of compliance with Dryad publication guidelines.

### Statistical analysis

After checking for normality, all continuous variables obeyed a skewed distribution and were therefore expressed as median (quartile) and compared by using the Mann-Whitney *U* test. For categorical variables, the data were described as frequencies (percentages) and were evaluated utilizing a chi-square test. All participants were divided equally into 4 groups according to the quartiles of AST/ALT ratio, including Q1 group (<0.89), Q2 group (0.89-1.18), Q3 group (1.18-1.53), and Q4 group (>1.53). Then, Kaplan-Meier curves were applied to describe the cumulative incidence of diabetes. In addition, Cox proportional hazards regression was performed to investigate the association between AST/ALT ratio and diabetes risk. Also, three adjusted models were constructed to reduce bias. Model 1 was adjusted for age, gender, BMI, and family history of diabetes. Model 2 was further adjusted for systolic blood pressure (SBP), diastolic blood pressure (DBP), FPG, cholesterol, triglycerides, high-density lipoprotein cholesterol (HDL-C), low-density lipoprotein cholesterol (LDL-C), blood urea nitrogen (BUN) and endogenous creatinine clearance rate (CCR) on the basis of model 1. Model 3 was further adjusted for the smoking status and drinking status on the basis of model 2. Finally, restricted cubic spline (RCS) with 4 knots based on model 3 were used to explore whether there was a non-linear correlation between the AST/ALT ratio and the risk of diabetes.

All statistical analyses were performed with SPSS (version 26.0) and R software (version 4.0.5). A *P*-value < 0.05 (two-sided) was considered statistically significant.

## Results

A total of 87,883 participants were eventually recruited into the study, of whom 1,877 developed diabetes during a median follow-up period of 3.01 years, accounting for 2.14%. Compared to participants without diabetes, the diabetes group was older and had a higher proportion of males and family history of diabetes. Also, the diabetes group contained a larger population of current smokers and drinkers. In addition, participants developing diabetes had higher baseline values of BMI, SBP, DBP, FPG, cholesterol, triglycerides, LDL-C, BUN, CCR, ALT, and AST, but lower HDL-C values. Notably, the AST/ALT ratio was only 0.96 in the diabetes group, which was lower than the 1.19 in the non-diabetes group, showing a statistically significant difference (Table 1).

**Table 1:** Baseline characteristics of the study participants.

**Variables**	**Total (*n* = 87,883)**	**Diabetes (*n* = 1,877)**	**Non-diabetes (*n* = 86,006)**	***P*** **value**
Age (year)	38.00 (32.00, 50.00)	55.00 (45.00, 63.00)	38.00 (32.00, 50.00)	< 0.001
Male, *n* (%)	50,090 (57.00)	1,321 (70.38)	48,769 (56.70)	< 0.001
BMI (kg/m^2^)	23.04 (20.82, 25.39)	25.91 (23.84, 28.10)	22.98 (20.78, 25.31)	< 0.001
SBP (mmHg)	118.00 (107.00, 129.00)	131.00 (119.00, 143.00)	118.00 (107.00, 129.00)	< 0.001
DBP (mmHg)	73.00 (66.00, 81.00)	80.00 (73.00, 88.00)	73.00 (66.00, 81.00)	< 0.001
FPG (mmol/L)	4.93 (4.56, 5.30)	6.04 (5.46, 6.47)	4.91 (4.55, 5.29)	< 0.001
Cholesterol (mmol/L)	4.60 (4.05, 5.22)	4.97 (4.31, 5.63)	4.60 (4.05, 5.21)	< 0.001
Triglyceride (mmol/L)	1.06 (0.72, 1.60)	1.70 (1.15, 2.49)	1.05 (0.71, 1.60)	< 0.001
HDL-C (mmol/L)	1.34 (1.17, 1.55)	1.30 (1.11, 1.52)	1.35 (1.17, 1.55)	< 0.001
LDL-C (mmol/L)	2.70 (2.29, 3.16)	2.84 (2.38, 3.29)	2.70 (2.29, 3.15)	< 0.001
BUN (mmol/L)	4.56 (3.83, 5.40)	4.91 (4.07, 5.80)	4.55 (3.82, 5.40)	< 0.001
CCR (umol/L)	70.90 (59.00, 82.00)	73.00 (63.00, 83.00)	70.70 (59.00, 82.00)	< 0.001
ALT (U/L)	18.00 (13.00, 27.80)	26.00 (18.00, 40.00)	18.00 (13.00, 27.30)	< 0.001
AST (U/L)	22.00 (18.60, 26.70)	25.30 (21.00, 32.10)	22.00 (18.50, 26.50)	< 0.001
AST/ALT	1.18 (0.89, 1.53)	0.96 (0.73, 1.25)	1.19 (0.89, 1.53)	< 0.001
***P*** **for trend**				< 0.001
Q1		793 (42.25)	21,177 (24.62)	
Q2		542 (28.88)	21,429 (24.92)	
Q3		317 (16.89)	21,654 (25.18)	
Q4		225 (11.99)	21,746 (25.28)	
**Smoking status**, ***n*** **(%)**				< 0.001
Current smoker	4,188 (19.01)	141 (32.56)	4,047 (18.74)	
Ever smoker	995 (4.52)	32 (7.39)	963 (4.46)	
Never smoker	16,843 (76.47)	260 (60.05)	16,583 (76.80)	
**Drinking status, n (%)**				< 0.001
Current drinker	581 (2.64)	23 (5.31)	558 (2.58)	
Ever drinker	3,837 (17.42)	85 (19.63)	3,752 (17.38)	
Never drinker	17,608 (79.94)	325 (75.06)	17,283 (80.04)	
Family history of diabetes, *n* (%)	1,772 (2.02)	71 (3.78)	1,701 (1.98)	< 0.001

ALT, alanine aminotransferase; AST, aspartate transaminase; BMI, body mass index; BUN, blood urea nitrogen; CCR, endogenous creatinine clearance rate; DBP, diastolic blood pressure; FPG, fasting plasma glucose; HDL-C, high-density lipoprotein cholesterol; LDL-C, low-density lipoprotein cholesterol; SBP, systolic blood pressure.

**Figure 2:**
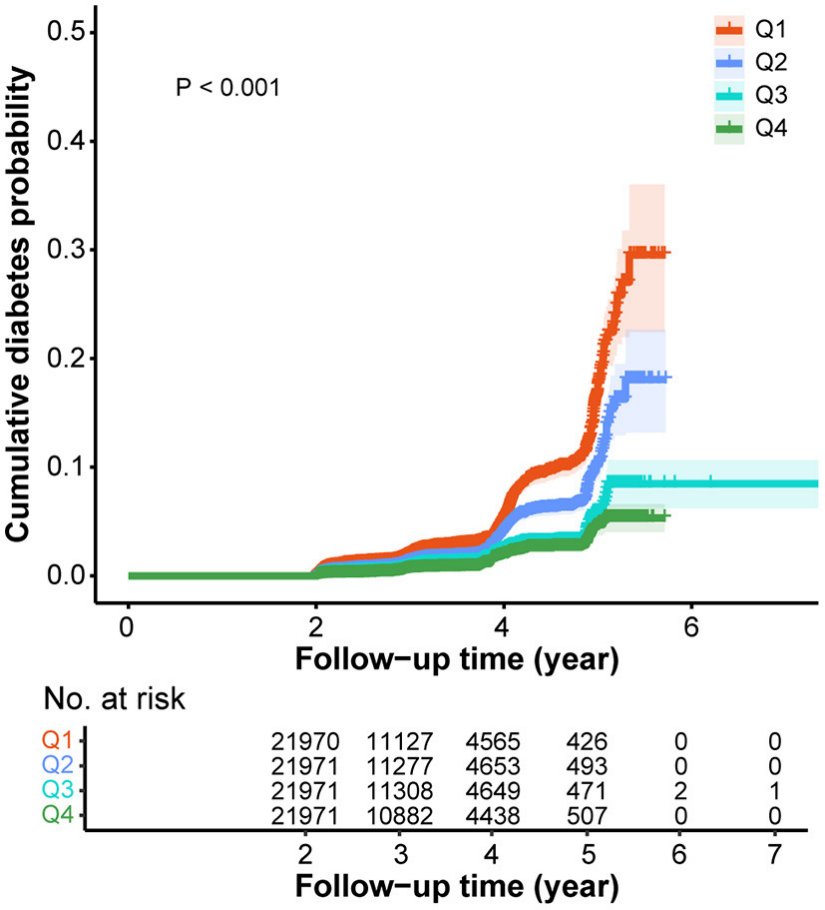
Kaplan-Meier curves for the cumulative diabetes probability according to different ASL/ALT (DeRitis) ratio. Q1, < 0.89; Q2, 0.89-1.18; Q3, 1.18-1.53; Q4, >1.53. ALT, alanine aminotransferase; AST, aspartate transaminase.

Then, we divided AST/ALT ratio into 4 levels based on quartiles and it was evident that 42.25% of participants in the diabetes group were in the Q1 group, much higher than the 24.62% in the non-diabetes group (Table 1). Meanwhile, Kaplan-Meier analysis was utilized to explore the effect of AST/ALT ratio on the cumulative probability of diabetes. It was observed that lower values of AST/ALT ratio were associated with a higher diabetes risk (*P* < 0.001) (Figure 2).

Next, four Cox proportional hazards regression models were constructed to analyze the independent effect of AST/ALT ratio in the risk of diabetes. In the initial unadjusted model, an increase in AST/ALT ratio was shown to reduce the risk of diabetes (HR: 0.32, 95% CI: 0.29–0.36, *P* < 0.001). The diabetes risk was 3.54 times higher in the low level Q1 group compared to Q4 group (95% CI: 3.05–4.10, *P* < 0.001). Models 1 and model 2, which adjusted for partial covariates, showed similar results. After further adjustment for smoking and drinking status, although no association was observed between quartiles of AST/ALT ratio and diabetes incidence, an increase in AST/ALT ratio still contributed to a reduced risk of diabetes (HR: 0.56, 95% CI: 0.37–0.85, *P* = 0.006) (Table 2).

**Table 2:** Univariate and multivariate Cox proportional hazards regression analyses for the association between AST/ALT (DeRitis) ratio and incident diabetes in different models.

**Variables**	**Univariate analysis**		**Multivariate analysis**					
			**Model 1**		**Model 2**		**Model 3**	
	**HR (95% CI)**	***P*** **value**	**HR (95% CI)**	***P*** **value**	**HR (95% CI)**	***P*** **value**	**HR (95% CI)**	***P*** **value**
AST/ALT	0.32 (0.29, 0.36)	< 0.001	0.32 (0.28, 0.37)	< 0.001	0.49 (0.41, 0.59)	< 0.001	0.56 (0.37, 0.85)	0.006
P for trend		< 0.001		< 0.001		< 0.001		^a^
Q1	3.54 (3.05, 4.10)	< 0.001	3.13 (2.66, 3.68)	< 0.001	1.82 (1.49, 2.23)	< 0.001		
Q2	2.33 (2.00, 2.72)	< 0.001	1.85 (1.58, 2.17)	< 0.001	1.27 (1.05, 1.54)	0.012		
Q3	1.37 (1.15, 1.62)	< 0.001	1.18 (1.00, 1.41)	0.055	0.85 (0.70, 1.05)	0.133		
Q4	Ref		Ref		Ref			

Model 1 was adjusted for age, gender, BMI and family history of diabetes. Model 2 was further adjusted for SBP, DBP, FPG, Cholesterol, Triglyceride, HDL-C, LDL-C, BUN and CCR on the basis of model 1. Model 3 was further adjusted for smoking status and drinking status on the basis of model 2.

a Represents *P* value > 0.05 and AST/ALT (DeRitis) ratio was not included in the final fitting model. ALT, alanine aminotransferase; AST, aspartate transaminase; BMI, body mass index; BUN, blood urea nitrogen; CCR, endogenous creatinine clearance rate; CI, confidence interval; DBP, diastolic blood pressure; FPG, fasting plasma glucose; HDL-C, high-density lipoprotein cholesterol; HR, hazard ratio; LDL-C, low-density lipoprotein cholesterol; SBP, systolic blood pressure.

**Figure 3:**
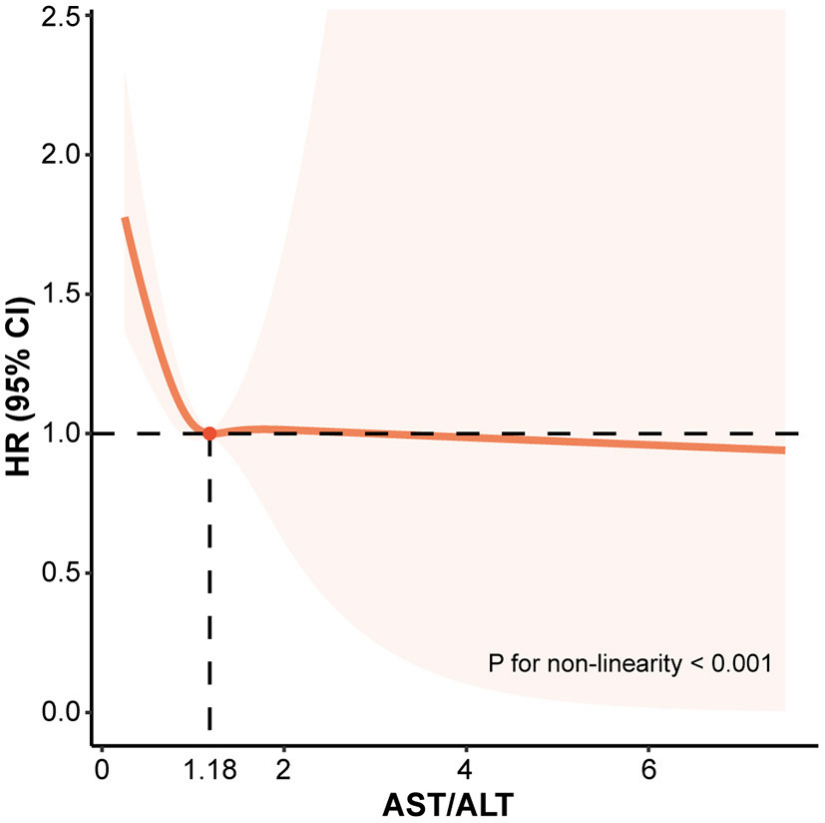
The association between ASL/ALT (DeRitis) ratio and the risk of diabetes assessed by restricted cubic splines analysis. ALT, alanine aminotransferase; AST, aspartate transaminase; CI, confidence interval; HR, hazard ratio.

Finally, RCS analysis based on model 3 was performed in order to visualize the association between AST/ALT ratio and diabetes risk. It was evident that there was a non-linear correlation between AST/ALT ratio and diabetes risk (*P* for non-linearity < 0.001). In the condition of AST/ALT ratio was ≤1.18, the risk of diabetes increased sharply as the AST/ALT ratio decreased. When the AST/ALT ratio was above 1.18, the diabetes risk was relatively stable (Figure 3). Further Cox regression analysis also showed that the diabetes risk increased as AST/ALT ratio decreased for AST/ALT ratios ≤1.18 (HR: 0.42, 95% CI: 0.19–0.91, *P* = 0.028). However, AST/ALT ratio did not independently affect the development of diabetes when it exceeded 1.18 (Table 3).

**Table 3:** Association between AST/ALT (DeRitis) ratio and incident diabetes based on restricted cubic spline regressions.

**Variables**	**Univariate analysis**		**Multivariate analysis**					
			**Model 1**		**Model 2**		**Model 3**	
	**HR (95% CI)**	***P*** **value**	**HR (95% CI)**	***P*** **value**	**HR (95% CI)**	***P*** **value**	**HR (95% CI)**	***P*** **value**
AST/ALT ≤ 1.18	0.27 (0.21, 0.36)	< 0.001	0.17 (0.13, 0.23)	< 0.001	0.21 (0.15, 0.32)	< 0.001	0.42 (0.19, 0.91)	0.028
AST/ALT > 1.18	0.67 (0.52, 0.86)	0.002	0.76 (0.59, 0.97)	0.025		^a^		^a^

Model 1 was adjusted for age, gender, BMI and family history of diabetes. Model 2 was further adjusted for SBP, DBP, FPG, Cholesterol, Triglyceride, HDL-C, LDL-C, BUN and CCR on the basis of model 1. Model 3 was further adjusted for smoking status and drinking status on the basis of model 2.

a Represents *P* value > 0.05 and AST/ALT (DeRitis) ratio was not included in the final fitting model. ALT, alanine aminotransferase; AST, aspartate transaminase; BMI, body mass index; BUN, blood urea nitrogen; CCR, endogenous creatinine clearance rate; CI, confidence interval; DBP, diastolic blood pressure; FPG, fasting plasma glucose; HDL-C, high-density lipoprotein cholesterol; HR, hazard ratio; LDL-C, low-density lipoprotein cholesterol; SBP, systolic blood pressure.

## Discussion

The large retrospective cohort study of 87,883 Chinese adults demonstrated that AST/ALT (DeRitis) ratio was a valuable predictor of diabetes. In addition, there was a non-linear correlation between AST/ALT ratio and diabetes risk. When AST/ALT ratio ≤1.18, the risk of diabetes increased rapidly along with a decrease in AST/ALT ratio. However, after AST/ALT ratio exceeded 1.18, AST/ALT ratio did not independently affect the development of diabetes.

The morbidity number of diabetes has increased quickly in the last few decades and diabetes has become the 9th leading cause of death (19). The factors associated with the development of diabetes are complex and involve multiple aspects such as genetic phenotypes, environmental exposures and lifestyle. In recent years, NAFLD has been recognized as an important risk factor for the development and progression of diabetes (20). In fact, the two diseases are mutually reinforcing (21). A global survey showed the prevalence of NAFLD to be over 55% in patients with type 2 diabetes (22). And another large Meta-analysis demonstrated that NAFLD was significantly associated with a 2–fold increased risk of diabetes (23). It is well–known that the liver plays an important role in maintaining blood glucose level and insulin homeostasis. Increased hepatic lipogenesis is strongly associated with an increased risk of insulin resistance and elevated fasting blood glucose, while insulin resistance further promotes the accumulation of fat in liver, thus leading to a vicious circle (5, 24).

ALT, AST, and GGT are important markers of liver function that have been shown to be associated with the risk of NAFLD (25). Recently, numerous studies have further explored the relationship between liver enzymes and diabetes. Most of the studies concluded that liver damage represented by elevated ALT and GGT levels increased the incidence of diabetes (26). High ALT values are related to hepatic insulin resistance and could prospectively predict decreased hepatic insulin sensitivity and the development of type 2 diabetes (27). The major controversy is found in the association between AST and diabetes. Numerous studies reported the favorable value of AST in predicting the incidence of diabetes, but a considerable number of studies also demonstrated no significant relationship between them (27–29). A study based on Chinese population also showed that ALT, but not AST, was associated with incident diabetes (7). Regardless, the positive results of various studies imply that liver enzymes may be a potential tool for assessing diabetes risk.

Recently, AST/ALT ratio and ALT/AST ratio have received a lot of attention and have been proved to be associated with a variety of diseases. A population-based longitudinal research showed that elevated ALT/AST ratio was related to the risk of new-onset NAFLD (10). Furthermore, ALT/AST ratio was more accurate in identifying hepatic steatosis than either ALT or AST alone (30). AST/ALT ratio was also reported to be closely related to metabolic syndrome (31). In the Korean population, a high ALT/AST ratio determined insulin resistance and obesity in adults (14). Similarly, surveys based on American populations revealed an increased risk of insulin resistance with lower AST/ALT ratio (32). Since insulin resistance is an important characteristic of diabetes, several recent studies based on Japanese populations also further elucidated a conclusion that there was also a correlation between low AST/ALT ratio and the incidence of diabetes (15, 16, 33). And they demonstrated that AST/ALT ratio was non-linearly associated with diabetes risk. However, diabetes risk scores developed on the basis of specific ethnicities might not be applicable to other ethnic groups (34). Thus, the inflection point of 1.18 was apparently higher in the Chinese population compared to the previous studies that explored an inflection point of 0.93 and 0.88 in the Japanese population (16, 33).

Many diabetes could be prevented by lifestyle improvements, such as maintaining a healthy weight and diet and increasing exercise, thus necessitating early identification of populations at high risk for early preventive measures (19). In fact, a number of novel biomarkers have been identified in recent years that are associated with the incidence of diabetes, such as branched amino acids, aromatic amino acids, phospholipids and hexose (35). Liver enzymes, which have been recommended by many researchers, are certainly one of the most cost-effective and convenient markers. It should still be emphasized that although the results of this study suggested that AST/ALT ratio could predict the risk of diabetes, however, AST and ALT themselves are not causative factors of diabetes based on the available studies, and diabetes are essentially achieved through liver function impairment. In addition, there are many factors that can lead to liver injury with elevated ALT and AST, including chronic alcohol consumption, NAFLD, viral infections, and drugs (such as non-steroidal anti-inflammatory drugs, cholesterol-lowering agents, and anti-tuberculosis drugs) (36). Therefore, we believe that it is essential to screen populations with low AST/ALT ratio in China to identify specific causes for diabetes prevention measures.

There were still some limitations to this study. Firstly, it was a secondary analysis study based on a public database. The original data obtained had already been screened, making the remaining data possibly not a good representation of the overall sample. In addition, the original cohort participants were mainly from southern Chinese cities, and further analyses will need to recruit more northern populations. Secondly, this was a retrospective study and even though we adjusted for multiple covariates, we could not neglect the influence of other unknown confounding factors on the results. Thirdly, numerous indicators, including AST and ALT, were only obtained at the time of initial examination, resulting in no possibility to analyze the influence due to fluctuating changes in covariates during the follow-up period. Further large prospective cohort studies are necessary in the future to elucidate the correlation between AST/ALT (DeRitis) ratio and diabetic events in the Chinese population.

## Conclusion

The AST/ALT (DeRitis) ratio is a valuable predictor of diabetes for Chinese populations and there is a non-linear correlation between them. In the future, medical practitioners are advised to provide timely diabetes prevention guidance for individuals with AST/ALT ratios below 1.18.

## Data availability statement

Publicly available datasets were analyzed in this study. This data can be found here: Chen et al. (18), https://doi.org/10.5061/dryad.ft8750v.

## Ethics statement

This study obtained ethical exemption from the Ethics Committee of Shanghai Tenth People's Hospital because of compliance with Dryad publication guidelines.

## Author contributions

WX, WY, and SC analyzed and interpreted the data and drafted the manuscript. WX and ZM collected and assembled the raw data. ZS and TY contributed to design and critically revised the manuscript for important intellectual content. All authors have read and agreed to the published version of the manuscript.

## Conflict of interest

The authors declare that the research was conducted in the absence of any commercial or financial relationships that could be construed as a potential conflict of interest.

## Publisher's note

All claims expressed in this article are solely those of the authors and do not necessarily represent those of their affiliated organizations, or those of the publisher, the editors and the reviewers. Any product that may be evaluated in this article, or claim that may be made by its manufacturer, is not guaranteed or endorsed by the publisher.

## Abbreviations

ALT: alanine aminotransferase
AST: aspartate aminotransferase
BMI: body mass index
BUN: blood urea nitrogen
CCR: endogenous creatinine clearance rate
CI: confidence interval
DBP: diastolic blood pressure
FPG: fasting plasma glucose
GGT: gamma-glutamyltransferase
HDL-C: high-density lipoprotein cholesterol
HR: hazard ratio
LDL-C: low-density lipoprotein cholesterol
NAFLD: non-alcoholic fatty liver disease
RCS: restricted cubic spline
SBP: systolic blood pressure.
